# Integration of computational modeling with membrane transport studies reveals new insights into amino acid exchange transport mechanisms

**DOI:** 10.1096/fj.14-267773

**Published:** 2015-03-11

**Authors:** Kate L. Widdows, Nuttanont Panitchob, Ian P. Crocker, Colin P. Please, Mark A. Hanson, Colin P. Sibley, Edward D. Johnstone, Bram G. Sengers, Rohan M. Lewis, Jocelyn D. Glazier

**Affiliations:** *Maternal and Fetal Health Research Centre, Institute of Human Development, University of Manchester, Manchester, United Kingdom; ^†^St. Mary's Hospital and Central Manchester University Hospitals National Health Service Foundation Trust, Manchester Academic Health Science Centre, Manchester, United Kingdom; ^‡^Bioengineering Science Research Group, Faculty of Engineering and the Environment, University of Southampton, Southampton, United Kingdom; ^§^Mathematical Institute, Oxford University, Oxford, United Kingdom; and ^¶^Faculty of Medicine, and ^‖^Institute for Life Sciences, University of Southampton, Southampton, United Kingdom

**Keywords:** antiporters, facilitated transport, LAT2 (*SLC7A8*), overshoot phenomena

## Abstract

Uptake of system L amino acid substrates into isolated placental plasma membrane vesicles in the absence of opposing side amino acid (zero-*trans* uptake) is incompatible with the concept of obligatory exchange, where influx of amino acid is coupled to efflux. We therefore hypothesized that system L amino acid exchange transporters are not fully obligatory and/or that amino acids are initially present inside the vesicles. To address this, we combined computational modeling with vesicle transport assays and transporter localization studies to investigate the mechanisms mediating [^14^C]l-serine (a system L substrate) transport into human placental microvillous plasma membrane (MVM) vesicles. The carrier model provided a quantitative framework to test the 2 hypotheses that l-serine transport occurs by either obligate exchange or nonobligate exchange coupled with facilitated transport (mixed transport model). The computational model could only account for experimental [^14^C]l-serine uptake data when the transporter was not exclusively in exchange mode, best described by the mixed transport model. MVM vesicle isolates contained endogenous amino acids allowing for potential contribution to zero-*trans* uptake. Both L-type amino acid transporter (LAT)1 and LAT2 subtypes of system L were distributed to MVM, with l-serine transport attributed to LAT2. These findings suggest that exchange transporters do not function exclusively as obligate exchangers.—Widdows, K. L., Panitchob, N., Crocker, I. P., Please, C. P., Hanson, M. A., Sibley, C. P., Johnstone, E. D., Sengers, B. G., Lewis, R. M., Glazier, J. D. Integration of computational modeling with membrane transport studies reveals new insights into amino acid exchange transport mechanisms.

Transport of amino acids across an epithelium is a complex process mediated by a broad array of amino acid transporters expressed in the plasma membrane (1, 2). These various transport proteins are classified according to their transport mechanism (2, 3). Accumulative transporters mediate transport of amino acids against an amino acid concentration gradient using secondary active transport dependent on electrochemical gradients. Obligate exchangers (antiporters) transfer one extracellular amino acid in exchange for an intracellular amino acid, whereas facilitated transporters (uniporters) enable facilitated diffusion down the prevailing amino acid concentration gradient (4, 5). The interaction of all 3 mechanisms is required for net directional transfer of amino acids across absorptive epithelia such as intestine, kidney, and placenta (5, 6).

Although these systems have been characterized at the molecular level (2, 4), their functional coordination and interaction in mediating transepithelial transport is poorly defined. We previously applied computational modeling to construct an integrated amino acid transport model for several amino acids across the human placental syncytiotrophoblast as a model epithelium (7). However, a more mechanistic modeling approach is required to fully understand the functional roles of the different amino acid transporters (8). Computational models can therefore be applied to generate simulations of specific transport mechanisms, and when validated with experimental data, can provide functional insight into amino acid transport activity not available through observation alone.

Among the various transport systems, system L is the principal Na^+^-independent transport system for providing cells with neutral amino acids, many of which are nutritionally essential. The 2 l-type amino acid transporters, LAT1 (*SLC7A5*) and LAT2 (*SCL7A8*) (9–11), are expressed in a variety of tissues such as the brain, kidney, gastrointestinal tract, testis, and placenta, indicating their key functional role in cellular growth and amino acid metabolism (12–14). System L transporters are reported to function as obligate exchangers, transporting one amino acid across the plasma membrane in direct exchange for another, thereby altering the relative composition of amino acids without affecting net amount (12, 15). Principal evidence for their obligatory exchange behavior stems from functional studies demonstrating that *trans*-stimulation of amino acid uptake can be induced by amino acid preloading (9, 15–17). However, there is inconsistency regarding the obligatory nature of the LAT2 subtype, which has been reported to exhibit a facilitated transport component (18, 19). This is of particular interest as we and others have previously reported the uptake of various system L substrates into non-preloaded plasma membrane vesicles (zero-*trans* uptake) isolated from the human placenta, which is incompatible with the principal of obligatory exchange where amino acids are required on both sides of the plasma membrane (16, 17, 20–22). These observations therefore led us to hypothesize that system L amino acid exchange transporters (*e.g.,* LAT2) may not be fully obligatory as previously thought. Alternatively, endogenous amino acids may be intrinsically present inside plasma membrane vesicles sufficient to enable obligatory exchange.

To address these hypotheses, we recently developed a computational amino acid transport model that describes the possibility for the uptake of l-serine (a LAT2 substrate) into human placental microvillous plasma membrane (MVM) vesicles by both obligate exchange and nonobligate transport mechanisms (*i.e.,* facilitated transport) (**Fig. 1*A***) (19). However, the previous experiments were unable to distinguish the extent to which these 2 transport mechanisms played a role. Therefore, our current aim was to design new experiments to further test the transport model and generate computational simulations to predict the transport mechanisms that best describe the uptake of l-serine into MVM vesicles. The biologic accuracy of the simulations was validated by determining how closely the model predictions for both transport mechanisms fitted the experimental data. In addition, we investigated the presence of endogenous amino acids in MVM vesicle isolates and explored how different exogenous preloaded amino acids affect l-serine uptake capacity. Finally, we examined the distribution of the LAT2 protein, and its localization with respect to LAT1, within the MVM of the human placental epithelial exchange barrier, the syncytiotrophoblast (SCT).

**Figure 1.**
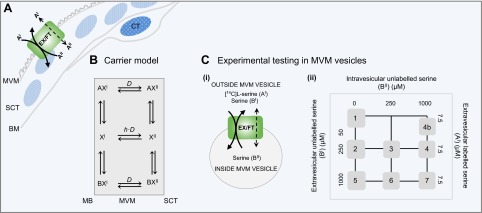
Experimental design for modeling system L amino acid transport across the MVM of human placenta. *A*) Schematic representation of the mode of system L amino acid transport across MVM comprising a capacity to function both as an obligate antiporter EX, as well as a facilitated transporter as defined by the carrier model. A^I^ and A^II^ represent tracer amino acid at the external face of MVM and in the cytoplasmic compartment of the SCT, respectively. *B*) The computational model was applied to describe the mixed transport carrier model with exchanger and facilitated transporter capacities as illustrated in *A* (19). For obligate exchange, transporter X can bind to either radiolabeled tracer amino acid A or an unlabeled amino acid B to form a complex AX^I^ or BX^I^, respectively, which can translocate between the external facing aspect of MVM in direct contact with maternal blood (MB) to the inside face of MVM in contact with the cytoplasm of SCT, represented as AX^II^ or BX^II^, respectively. In contrast, transporter X can also translocate between the external and internal faces unloaded, depending on the parameter *h*, which describes the rate at which the unbound transporter (X^I^ or X^II^) can translocate back across the membrane relative to the rate of amino acid transporter binding (*D*). The case *h* = 0 corresponds to an obligatory exchanger, whereas for *h* > 0, the transporter will display unidirectional facilitative transport governed by the direction of the transmembrane amino acid concentration gradient. *C*) The computational model was tested experimentally by measuring the Na^+^-independent uptake of [^14^C]l-serine (A^I^) (tracer) into isolated human placental MVM vesicles in response to various intravesicular (B^II^) and extravesicular (B^I^) additions of unlabeled l-serine (*Ci*) outlined in the experimental matrix in *Cii*. CT, cytotrophoblast cell; EX/FT, exchanger/facilitated transporter.

## MATERIALS AND METHODS

### Mathematical model of amino acid exchange transporters

We recently described a computational model of system L transport that specifically addresses the possibility for obligate and nonobligate exchange (corresponding to a facilitated transport component; Fig. 1*B*) (19). The proposed carrier-mediated model assumes that amino acid must bind to the transporter (carrier) to confer transport across the plasma membrane. Once bound, the transporter can undergo a conformational change exposing the substrate binding site to the opposite side of the plasma membrane, thereby permitting translocation.

The obligate exchange model assumes bidirectional transport of amino acids across the plasma membrane with 1:1 stoichiometry, for example*.,* amino acid must be present on both sides of the plasma membrane to confer transport (antiport) (Fig. 1*B*). In contrast, the transporter can also function with varying degrees of facilitated transport whereby the unidirectional transport of amino acids across the plasma membrane is driven by the direction of the transmembrane amino acid concentration gradient. The 2 transport mechanisms are distinguished by the parameter *h*, which defines the relative mobility of the unbound transporter (*i.e.,* the rate of transition of the unbound transporter relative to the translocation rate of the bound amino acid-transporter complex). The case *h* = 0 corresponds to obligate exchange, whereas for *h* > 0, the transporter will display varying degrees of facilitated transport. Thus, the carrier model was hypothesized to represent a single transporter that permits substrate transport by either (1) obligate exchange or (2) nonobligate exchange with a facilitated transport component (mixed transport model). Both transport models express net transport as a function of transporter mobility (translocation rate *D*), substrate binding (dissociation constant *K*), and the concentrations of amino acid on both sides of the plasma membrane. A detailed description of the underlying model assumptions and formulations is given in Panitchob *et al.* (19).

### Experimental testing using isolated plasma membrane vesicles

The 2 hypothesized transport mechanisms were tested experimentally using plasma membrane vesicles isolated from the maternal-facing MVM of human placenta. This epithelial plasma membrane can be isolated in relatively pure form and is extensively used to characterize various amino acid transport systems (23–27). Radiolabeled l-serine was selected as the amino acid substrate because of its preference for LAT2 over LAT1 (7, 16).

To separate the 2 transport mechanisms mediating l-serine uptake into MVM vesicles, we developed a specific set of experimental protocols designed to exploit the underlying transport characteristics of obligate and facilitated transport, as defined in the computational model hypotheses (Fig. 1*B*). Because both obligate exchangers and facilitated transport phenomena are intrinsically dependent on the concentration of amino acids on both sides of the membrane, we measured the uptake of [^14^C]l-serine (tracer) into isolated MVM vesicles in response to variations in intravesicular and extravesicular unlabeled l-serine concentrations, outlined in the experimental matrix (Fig. 1*C*). In essence, these conditions were used to test the capacity of the exchanger (antiporter) to be *trans*-stimulated, together with its ability to act as a facilitated transporter (uniporter) in the presence of a transmembrane amino acid concentration gradient. In particular, the 2 transport mechanisms were postulated to be differentially affected by added l-serine concentrations inside the MVM vesicles, leading to different concentrations of [^14^C]l-serine inside the vesicles at equilibrium. The obligatory exchange mechanism was predicted to equilibrate buffer composition through 1:1 bidirectional exchange resulting in the same proportion, but different absolute levels of [^14^C]l-serine, dependent on the concentration of l-serine added inside the vesicle. In contrast, in the case of the mixed transport model, [^14^C]l-serine influx would eventually reach an equilibrium concentration that would be identical across all experimental conditions.

To test these hypotheses, [^14^C]l-serine uptake into MVM vesicles was measured in response to 3 main situations: (1): no (initial) internal l-serine added yet increasing external l-serine concentrations (zero-*trans* experiments 1, 2, and 5)—the inwardly directed l-serine gradient is predicted to drive facilitated transport as there is no initial internal l-serine to drive 1:1 obligatory exchange; (2) constant external l-serine concentration with increasing internal l-serine concentrations (experiments 2, 3, 4, and 4b)—the outwardly directed l-serine gradient is predicted to lead to *trans*-stimulation of exchange; and (3) high concentrations of external l-serine (experiments 5–7) to study the comparable effects of *trans*-stimulation of exchange and transporter saturation (Fig. 1*C*).

### Preparation of plasma membrane vesicles

Placentas were obtained following written informed consent with the approval of the Central Manchester Research Ethics Committee (REC 12/NW/0574) from uncomplicated singleton pregnancies at term (38–40 wk of gestation) delivered by Caesarean section. MVM vesicles were isolated using Mg^2+^ precipitation and differential centrifugation as described previously (23). The final MVM pellet was resuspended in intravesicular buffer (IVB; 290 mM sucrose, 5 mM 4-(2-hydroxyethyl)-1-piperazineethanesulfonic acid, and 5 mM Tris, pH 7.4) alone (experiments 1, 2, and 5) or preloaded with 250 (experiments 3 and 6) or 1000 *µ*M unlabeled l-serine (experiments 4, 4b, and 7). MVM fragments were vesiculated by passing 15 times through a 25-gauge needle and stored at −80°C prior to use. MVM protein concentration was determined using the Lowry method (28), and purity was assessed by measuring the enrichment of MVM alkaline phosphatase activity compared with the placental homogenate (23). MVM vesicles were 21.0 ± 1.4 times enriched compared with initial placental homogenate (means ± sem, *n* = 9 placentas).

### Na^+^-independent uptake of [^14^C]l-serine into MVM vesicles

Na^+^-independent uptake of 7.5 *µ*M [^14^C]l-serine (tracer; 0.1 *µ*Ci/*µ*l; Perkin Elmer, Beaconsfield, Buckinghamshire, United Kingdom) into MVM vesicles was measured at room temperature using rapid vacuum filtration (16, 25) corresponding to the conditions set by the experimental matrix (Fig. 1*C*). MVM vesicles (diluted to 10 mg/ml protein with IVB) were equilibrated to room temperature (21–25°C) prior to uptake. [^14^C]l-serine uptake was initiated by addition of 20 *µ*l MVM vesicle suspension to 20 *µ*l extravesicular buffer (5 mM 4-(2-hydroxyethyl)-1-piperazineethanesulfonic acid, 5 mM Tris, and 145 mM KCl, pH 7.4) containing 7.5 *µ*M [^14^C]l-serine without or with unlabeled l-serine (0, 250, or 1000 *µ*M). For experiments 4 and 4b, the high intravesicular l-serine concentration (1000 *µ*M) was diluted in either 50 or 380 *µ*l extravesicular buffer to generate an extravesicular l-serine concentration of 250 or 50 *µ*M, respectively. Uptake was measured at variable time points up to 10 min and stopped by the addition of 2 ml ice-cold Krebs buffer (130 mM NaCl, 10 mM Na_2_HPO_4_, 4.2 mM KCl, 1.2 mM MgSO_4_, and 0.75 mM CaCl_2_, pH 7.4) and filtered through a 0.45 *µ*M nitrocellulose filter under vacuum. Filters were washed with 10 ml Krebs buffer, and the filter-associated radioactivity was determined by liquid scintillation counting. No protein controls (replacement of MVM vesicle protein by IVB) were included in parallel to determine tracer binding to the filter, which was subtracted from total vesicle count.

### Model predictions

**Figure 2.**
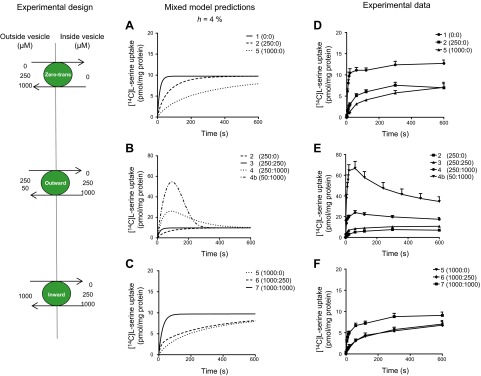
Computational simulations for the mixed transport model and corresponding experimental [^14^C]l-serine uptake data. *Left*) Schematic figure outlining the experimental design for model simulations, showing the imposed direction and magnitude of the transmembrane l-serine concentration gradients generated to drive uptake of tracer (7.5 *µ*M [^14^C]l-serine) into MVM vesicles (depicted as green circles). *A–C*) Computational simulations (with *h* set at 4%) for experiments 1, 2, and 5; experiments 2–4 and 4b; and experiments 5–7 of the experimental test matrix with varying substrate conditions (external: internal unlabeled l-serine concentration in micromolar). *A*) In the absence of added intravesicular l-serine to drive exchange, [^14^C]l-serine uptake was predicted to occur by facilitated transport as there is no added opposing amino acid to drive exchange (experiment 1). However, increases in the external l-serine concentration were predicted to decrease [^14^C]l-serine uptake in a concentration-dependent manner because of *cis*-inhibition (experiments 2 and 5). *B*) In contrast, the addition of l-serine inside the vesicles was predicted to *trans*-stimulate [^14^C]l-serine uptake (experiments 3, 4, and 4b). Interestingly, the outwardly directed concentration gradient in experiments 4 and 4b was predicted to lead to an overshoot curve (transient accumulation of [^14^C]l-serine above final equilibrium concentration). *C*) High extravesicular l-serine concentrations were predicted to reduce l-serine equilibrium concentrations compared with experiments 2, 3, and 4 because of *cis*-inhibition. *D–F*) Experimentally measured Na^+^-independent uptake of 7.5 *µ*M [^14^C]l-serine into human placental MVM vesicles unloaded or preloaded with l-serine closely resembled the model simulations shown in *A–C* (note the difference in axis scales). Symbol numbers relate to experimental condition given by the experimental matrix. Data are shown as means + sem (*n* = 5–9 placentas).

Model simulations were generated to predict the uptake of [^14^C]l-serine into MVM vesicles under the 8 experimental conditions according to the obligate exchange and mixed transport model (Fig. 2*A–C*). Model parameters were determined from previous [^14^C]l-serine uptake kinetics and zero-*trans* time course experiments (19). For the obligatory model, *h* was set to zero, whereas for the mixed transport model, the relative mobility of the unbound transporter parameter (*h*) was chosen to be 0.04, based on a previous report that estimated an upper confidence interval of 4% for efflux by facilitated transport relative to obligatory exchange (15). Based on this value of *h*, a dissociation constant (*K*) of 1129 *µ*M and fitted value of the transporter’s effective uptake rate (*V*) of 92 *µ*M/min were derived [detailed model equations are given in Panitchob *et al.* (19)]. Time course integration was performed to obtain estimated intravesicular concentrations from the model equation for tracer influx (19). An intravesicular volume of 1.3 *µ*l/mg protein was used to generate model predictions, based on the mean value obtained from previous experiments performed under similar buffer conditions (29, 30) (data not shown). All computational simulations were performed using MATLAB (matrix laboratory; MathWorks, Natick, MA, USA).

### Model fittings

The experimental [^14^C]l-serine uptake data (picomoles per milligram protein) were converted to a concentration (micromolar) as described above and fitted to the 2 distinct transport models using a least squares criterion. Three parameters were fitted for the mixed transport model: (1) the relative mobility of the unbound transporter *h*, (2) the effective uptake rate *V*, and (3) the dissociation constant *K.* Similarly, *V* and *K* were fitted for the obligatory model; however, in accordance with the obligatory model assumptions, *h* was set to zero, and instead a potential initial concentration of intravesicular l-serine was estimated that would enable exchange (19). The averaged data (*n* = 5–9 placentas) for all 8 conditions were fitted simultaneously based on a single set of parameters, rather than each experiment separately.

### *Trans*-stimulation of [^14^C]l-serine uptake by various intravesicular amino acids

Our initial experiments demonstrated that a steep outwardly directed transmembrane concentration gradient created by addition of intravesicular l-serine caused substantial *trans*-stimulation of 7.5 *µ*M [^14^C]l-serine influx into MVM vesicles (experiment 4b). To investigate the effect of other substrate amino acids/analogs, MVM vesicles were preloaded at the time of vesiculation with 1000 *µ*M l-serine, l-alanine, glycine, l-methionine, l-phenylalanine, l-leucine, 2-aminobicyclo[2.2.1]heptane-2-carboxylic acid, or d-leucine as potential system L substrates. In addition, l-proline and *α*-methylaminoisobutyric acid (MeAIB) were included as controls that were not system L substrates, and mannitol addition served as osmotic control. Uptake of 7.5 *µ*M [^14^C]l-serine into MVM vesicles was measured as described above.

### Endogenous amino acid concentration in MVM vesicles

To explore the possibility that zero-*trans* uptake might be explained by the presence of endogenous amino acids within MVM vesicles, amino acid concentrations within the vesicle isolates was measured by HPLC following deproteinization with sulphosalicylic acid.

### Cellular localization of LAT2 and LAT1 in human placenta

In line with our hypothesis for LAT2-mediated l-serine uptake, we examined the expression of LAT2 and its colocalization with LAT1 in human term placenta using immunofluorescence. Frozen villous sections (3 *µ*M) were postfixed in 10% neutral buffered formalin for 15 minutes. Tissues were permeabilized using 0.5% Triton X-100 diluted in Tris-buffered saline (100 mM Tris and 150 mM NaCl, pH 7.6) for 15 minutes at room temperature. To minimize autofluorescence, sections were incubated with sodium borohydrate (10 mg/ml) diluted in ice-cold Tris-buffered saline for 3 × 10 minutes at 4°C. Nonspecific protein binding sites were blocked using 10% normal donkey serum (Dako, Ely, United Kingdom) diluted in Tris-buffered saline for 30 minutes. Sections were incubated with either rabbit polyclonal anti-LAT1 (19 *µ*g/ml, P00801; Capralogics, Hardwick, MA, USA) for 1 hour at room temperature (31, 32) or mouse monoclonal anti-LAT2 (20 *µ*g/ml, clone 8B12; Abcam, Cambridge, United Kingdom) at 4°C overnight. Incubations with the same host species IgG served as negative controls. Sections were washed with Tris-buffered saline, and the antibody-antigen reaction was detected by incubation with donkey anti-rabbit Alexa Fluor 488-green and/or donkey anti-mouse Alexa Fluor 568-red diluted 1:500 in Tris-buffered saline for 1 hour at room temperature. Following washing with Tris-buffered saline, nuclei were visualized using DAPI (5 nM; Dako) incubated for 5 minutes and mounted with Dako mounting medium.

### Western blotting for LAT2 in MVM

Western blotting was performed to confirm expression of LAT2 and its associated heavy chain CD98 (C-20; Santa Cruz Biotechnology, Santa Cruz, CA, USA) (33) in MVM vesicles. Vesicles (40 *µ*g protein) were electrophoresed under reducing and nonreducing, nonboil conditions (8 M urea, 5% SDS, 0.4% bromophenol blue, 455 mM DTT, and 50 mM Tris-HCl, pH 6.9) using 3% stacking and 10% resolving polyacrylamide gels. Proteins were transferred to HyBond ECL nitrocellulose membranes. Membranes were blocked using 5% nonfat dried milk (Marvel) diluted in Tris-buffered saline-T (10 mM Tris-HCl, 150 mM NaCl, and 0.05% Tween-20, pH 8.0) for 1 hour, followed by incubation with either LAT2 (1 *µ*g/ml) or CD98 (0.5 *µ*g/ml) diluted in 5% nonfat dried milk in Tris-buffered saline-T for 1 hour at room temperature. Following washing with Tris-buffered saline-T for 3 × 5 minutes, membranes were incubated for 1 hour with horseradish peroxidase-conjugated anti-mouse IgG and anti-goat IgG secondary antibodies, respectively (Dako) diluted 1:2000 in 5% nonfat dried milk in Tris-buffered saline. Immunoreactivity was detected by ECL (Pierce, Rockford, IL, USA). Negative controls were performed by omission of primary antibody.

### Statistics

Statistical analyses were carried out using GraphPad Prism 5.0 (GraphPad Software, La Jolla, CA, USA). Between-group analyses were performed using a 2-way repeated-measures ANOVA followed by a Bonferroni *post hoc* test. Peak overshoot (60 seconds) *versus* decay (10 minutes) was analyzed using a paired *t* test. *P* < 0.05 was considered statistically significant. Data are presented as means + sem, with the number of experiments (*n*) denoting the number of placentas studied.

## Results

### Model predictions for [^14^C]l-serine uptake into MVM vesicles

Model simulations were generated to predict [^14^C]l-serine uptake into MVM vesicles by the 2 hypothesized transport mechanisms in response to the experimental conditions. For the mixed transport model (**Fig. 2*A***), the predicted outcomes for the zero-*trans* experiments (experiments 1, 2, and 5) displayed inhibition of the initial rate of [^14^C]l-serine uptake in response to increasing external unlabeled serine concentrations (*cis*-inhibition) while approaching the same equilibrium. The addition of intravesicular l-serine (experiments 3, 4, 4b, 6, and 7) was predicted to *trans*-stimulate [^14^C]l-serine uptake into MVM vesicles in a concentration-dependent manner, *i.e.,* higher internal concentrations of unlabeled l-serine led to a higher initial rate of [^14^C]l-serine uptake (Fig. 2*B, C*). Interestingly, the mixed model predicted that the presence of a high outwardly directed l-serine concentration gradient (experiments 4 and 4b) would lead to an initial overshoot of [^14^C]l-serine accumulation into MVM vesicles above equilibrium concentrations by a dominant fast exchange mechanism followed by attenuation to apparent equilibrium by a slower facilitated transport mechanism (Fig. 2*B*). For the obligate exchange model (predictions not shown), the predicted outcomes for zero-*trans* experiments (experiments 1, 2, and 5) in the absence of exogenous intravesicular l-serine were zero consistent with the concept of obligate exchange in which amino acids are required at both sides of the plasma membrane to confer transport. In contrast, the addition of internal concentrations of unlabeled l-serine was predicted to lead to the accumulation of [^14^C]l-serine at different equilibrium concentrations, proportional to the intravesicular concentration added (Fig. 2*C*). In contrast, increases in external serine concentrations were predicted to decrease the equilibrium concentration of [^14^C]l-serine proportionally.

### [^14^C]l-serine uptake into MVM vesicles corresponds to simulated uptake curves by the mixed transport model

Fig. 2*D–F* shows the uptake of [^14^C]l-serine into MVM vesicles under the conditions set by the experimental matrix (Fig. 1*C*). We observed time-dependent uptake of [^14^C]l-serine into MVM vesicles in the absence of exogenous intravesicular amino acid (experiments 1, 2, and 5), which is incompatible with the concept of obligatory exchange (Fig. 2*D*). In these cases, the rate of [^14^C]l-serine uptake significantly declined in the presence of extravesicular l-serine, confirming *cis*-inhibition of a carrier-mediated process (experiments 2 and 5). In contrast, addition of intravesicular l-serine significantly *trans*-stimulated [^14^C]l-serine uptake in a concentration-dependent manner (Fig. 2*E*), confirming that influx of extravesicular tracer is stimulated by the presence of intravesicular amino acid (experiment 2 *vs*. 4, *P* < 0.05; experiment 2 *vs*. 4b, *P* < 0.001; experiment 3 *vs*. 4, *P* < 0.001; experiment 3 *vs*.4b, *P* < 0.001). Notably, the degree of *trans*-stimulation depended on both the direction and magnitude of the transmembrane serine concentration gradient (Fig. 2*E, F*). These experiments showed that the presence of a high outwardly directed transmembrane l-serine gradient led to the rapid accumulation of [^14^C]l-serine above equilibrium concentration at 60 s (overshoot curve; experiment 4, 23.9 ± 1.9 pmol/mg protein *vs*. experiment 2, 5.1 ± 0.5 pmol/mg protein; *P* < 0.001). Following peak accumulation, [^14^C]l-serine uptake subsequently declined with time reaching apparent equilibrium by 10 minutes (overshoot decay; 60 seconds *vs*. 10 minutes: experiment 4, *P* = 0.001; experiment 4b, *P* < 0.001). Noticeably, the peak of the overshoot was significantly enhanced by a steeper gradient (experiment 4b; Fig. 2*E*; *P* < 0.001 compared with experiments 4 and 2 with nonloaded vesicles). The overshoot curve was interpreted to represent [^14^C]l-serine uptake by an exchange mechanism coupled with a facilitated transport mechanism (as predicted by the mixed transport model). In these cases, initial uptake of [^14^C]l-serine occurs primarily by a fast exchange mechanism driven by the outwardly directed transmembrane l-serine gradient (overshoot peak). The subsequent decline in [^14^C]l-serine uptake between 60 seconds (peak) and 10 minutes was inferred to represent the (relatively slower) efflux of [^14^C]l-serine by a facilitated transport mechanism (overshoot decay). Conversely, the presence of an inwardly directed l-serine concentration gradient did not elicit overshoot above equilibrium (Fig. 2*D*). All uptakes were linear up to 20 seconds (data not shown). The contribution of vesicle swelling to the overshoot was excluded because changes in intravesicular volume during the experiment were negligible (data not shown).

### Carrier model fittings to experimental data

**Figure 3.**
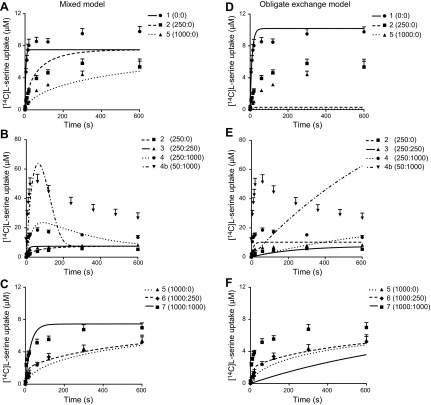
Carrier model fittings to experimental data demonstrate that l-serine uptake is best described by the mixed transport model and not by obligate exchange (*A*–*F*). The experimental data were converted to concentration (micromolar) based on an intravesicular volume of 1.3 *µ*l/mg protein and fitted to the carrier models. Fittings for the mixed model (*A*–*C*) and obligate exchange model (*D*–*F*) are shown as solid and dashed lines for the various experimental conditions. Symbol numbers relate to experimental condition given by the experimental test matrix with l-serine concentrations in parentheses shown as (extravesicular: intravesicular; in micromolar), respectively. Data are means + sem (*n* = 5–9 placentas).

The biologic accuracy of the simulated l-serine uptake curves was determined by fitting the experimental l-serine uptake data to the obligate exchange model and mixed transport model (**Fig. 3**). For each model, a single set of parameters was fitted. Overall, the experimental data were in poor agreement with model predictions for obligatory exchange across all 8 experimental conditions (Fig. 3*D–F*). To enable exchange for the zero-*trans* uptakes (Fig. 3*D*), the model fitted a low initial endogenous l-serine concentration inside the vesicle of 10 *µ*M; however, in doing so, it was only able to represent experiment 1 adequately while severely underpredicting experiments 2 and 5. In addition, the obligatory model was unable to represent the other experimental conditions (Fig. 3*E, F*). An *R*^2^ test revealed that the obligatory model was incapable of representing the data any better than a straight line through the mean of the data (*R*^2^ = −0.099). In contrast, the experimental data for [^14^C]l-serine uptake into MVM vesicles displayed good concordance to the simulations for the mixed transport model across all experimental conditions (Fig. 3*A–C*; *R*^2^ = 0.69). Most notably, the predicted overshoot of [^14^C]l-serine uptake in response to an outwardly directed l-serine concentration gradient (experiments 4 and 4b) was indeed observed experimentally, and closely resembled the simulated overshoot by the mixed transport model (Fig. 3*B*); this was not observed for the obligatory exchange model (Fig. 3*E*). However, the simulated curve underestimated the experimental equilibrium concentrations, most notable in experiment 4b. The relative mobility of the unbound transporter parameter (*h*) was fitted to be 0.16, which implies the rate for facilitated transport relative to obligate exchange was slow at *h* = 16%.

### *Trans*-stimulation of [^14^C]l-serine uptake by a broad variety of intravesicular amino acids

**Figure 4.**
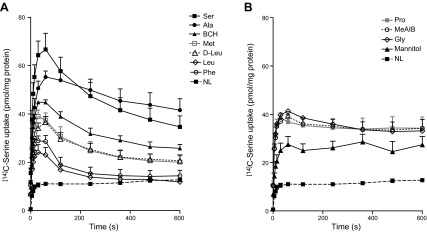
A high outwardly directed substrate concentration gradient imposed by various intravesicular system L amino acid substrates shows *trans*-stimulation of [^14^C]l-serine uptake resulting in overshoot phenomena. *A*) Various system L amino acid substrates or analogs were preloaded during vesiculation into MVM vesicles, and [^14^C]l-serine uptake was measured by incubation in Na^+^-free extravesicular buffer compared with nonloaded (NL) vesicles. *B*) To control for nonselective uptake effects, l-proline, MeAIB, and the osmotic control mannitol were also included. Data are means + sem (*n* = 4–9 placentas).

We determined the intravesicular amino acid selectivity of amino acid exchange transporters by comparing the *trans*-stimulation of [^14^C]l-serine uptake induced by various amino acids preloaded into MVM vesicles. **Fig. 4** shows the influence of a high outwardly directed substrate concentration gradient on the Na^+^-independent uptake of [^14^C]l-serine into MVM vesicles by various neutral amino acids and analogs. Preloading with 1000 *µ*M l-serine, l-alanine, 2-aminobicyclo[2.2.1]heptane-2-carboxylic acid (BCH), glycine, l-methionine, d-leucine, l-leucine, and l-phenylalanine, all known substrates of the system L transport system, resulted in the substantial and differential *trans*-stimulation of [^14^C]l-serine uptake above equilibrium concentration (overshoot at 60 seconds), followed by subsequent reduction with time (Fig. 4*A*), consistent with model predictions for the mixed transport model (Fig. 2*B*). Surprisingly, l-proline and MeAIB, both substrates of the Na^+^-dependent system A accumulative transporters, were similarly effective at stimulating [^14^C]l-serine uptake into MVM vesicles, whereas in contrast, they did not elicit loss of tracer with time (Fig. 4*B*). Hence, varying the intravesicular amino acid availability affects not only the magnitude of l-serine influx but also exchange capacity. Interestingly, none of the tested amino acids demonstrated a higher *trans*-stimulation than l-serine (sixfold increase in uptake), and likewise, the highest degree of *trans*-stimulation was seen for LAT2 specific substrates (l-serine, glycine, and l-alanine). With the exception of l-leucine, l-methionine, l-proline, and MeAIB, all uptakes were linear up to 15 seconds (data not shown).

### Endogenous amino acids are present in MVM vesicle isolates

**Figure 5.**
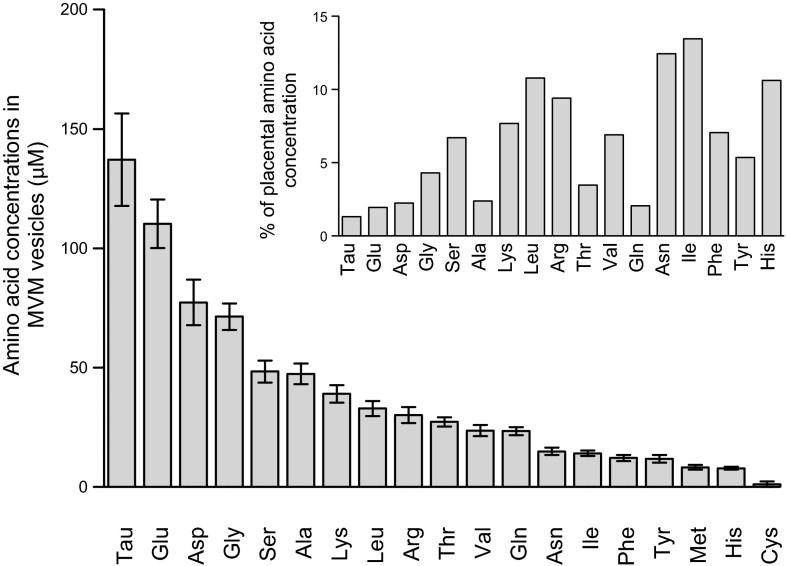
Endogenous amino acid concentrations in human placental MVM vesicle isolates. Amino acid concentrations in MVM vesicle isolates are shown in order of diminishing concentration. The relative ratio (as percentage) of amino acid concentration in MVM vesicle isolates to that reported previously in placental tissue is shown in the upper graph (34). Data are shown as means ± sem (*n* = 4 placentas).

Amino acids were found to be present in MVM vesicle isolates and are shown in order of decreasing concentration (**Fig. 5**). The relative concentration of amino acids found in MVM isolates follows a similar pattern and ranking to previous estimates of free intracellular amino acid concentrations in human placental tissue (34). However, the proportionality of amino acid concentrations found in MVM vesicle isolates to reported placental tissue concentrations was variable across different amino acids (Fig. 5, upper).

The potential impact of the measured endogenous amino acid concentrations on the model predictions was evaluated by repeating the model fits as in Fig. 3, but with an additional 300 *μ*M of substrate in the vesicle suspension reflecting total LAT2 substrate concentrations (Fig. 5), and applying the appropriate dilutions of external concentrations for each experiment (results not shown). As before, the obligatory exchange model could not represent the experimental data (*R*^2^ = −0.14). Notably for the nonobligatory transport model, *trans*-stimulation due to additional endogenous substrate led to an overshoot for the nominally zero-*trans* case (experiment 1). In addition, the results displayed a significant underprediction of initial rates and time delays in the peak of the overshoot, and overall fit quality was reduced (*R*^2^ = 0.49). This lack of correspondence to the experimental data indicates that not all of the measured endogenous substrate may be freely exchangeable.

### Colocalization of system L transporters LAT1 and LAT2

**Figure 6.**
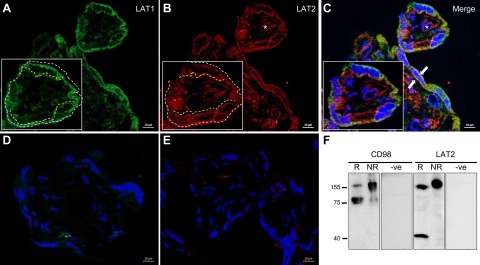
Immunolocalization of LAT2 to the MVM of human placental SCT and colocalization with LAT1. *A*–*C*) The l-type amino acid transport proteins LAT1 (green) and LAT2 (red) were expressed in both the maternal-facing MVM and fetal-facing BM of the human placental SCT, showing areas of colocalization (orange, arrows). However, the membrane distribution of the two proteins varied, such that LAT1 appeared to be more strongly associated with the MVM (white dotted line) compared with the BM (yellow dotted line), whereas in contrast, LAT2 appeared to be strongly expressed throughout both plasma membranes; LAT2 positivity was also observed within the fetal endothelium (asterisks). Relatively minor diffuse staining was seen in the SCT for both proteins. Staining was absent in matched isotype negative controls for LAT1 (*D*) and LAT2 (*E*). Blue = DAPI. Final magnification, ×630. *F*) Western blotting for LAT2 and its associated heavy chain CD98 in MVM membranes from term human placenta. Under nonreducing conditions (NR), LAT2 and CD98 antibodies showed immunoreactive species at ∼155 kDa, which were absent from the negative controls (−ve). Under reducing conditions (R), the ∼155 kDa LAT2-CD98 heterodimer was reduced to either an ∼40 kDa (LAT2) or ∼80 kDa (CD98) band. The observed mobility shift (9) thus confirms antibody specificity for the LAT2 protein in human placental MVM.

**Fig. 6*A–C*** shows the expression of the l-type amino acid transporters in human placental villi. Both LAT1 and LAT2 transport proteins were expressed on the maternal-facing MVM and fetal-facing basal plasma membrane (BM) of the placental epithelial transport barrier, the SCT. Staining showed a variable pattern with evidence for colocalization for LAT1 and LAT2 within both plasma membranes. Relatively minor diffuse foci were also observed throughout the SCT. Staining was absent in matched isotype negative controls (Fig. 6*D, E*).

### LAT2 is expressed in MVM of human placenta

The presence of the LAT2 protein in the (maternal-facing) MVM of the human placenta, linked to CD98, was confirmed by Western blotting (Fig. 6*F*). Under nonreducing conditions, both LAT2 and CD98 antibodies demonstrated immunoreactive species that comigrated at ∼155 kDa. However, a mobility shift from 155 (CD98-LAT2 heterodimer) to ∼40 (LAT2 monomer) and ∼80 kDa (CD98 glycoprotein) was observed under reducing conditions, respectively, thus validating the antibody specificity for the LAT2 protein. These bands were not present in the negative controls (omission of primary antibody).

## Discussion

This study presents a novel integrated approach that combines computational modeling with membrane transport studies to investigate the underlying mechanisms of amino acid exchange transporters. Our studies reveal that exchangers, believed to be obligatory, may exhibit other patterns of transport behavior relating to facilitated transport. This conclusion is inferred from the observation that the computational model could only account for the uptake of l-serine into MVM vesicles when the transporter did not function exclusively in exchange mode. Importantly, this delineation could not have been derived without computational simulation, which anticipated distinct differences in l-serine transport depending on the degree of exchange obligation. The integration of computational simulation and experimental validation has therefore led to new quantitative insights into amino acid exchange transport mechanisms, which could not have been derived from experimentation alone.

As demonstrated, the principal strength of the model was the ability to distinguish and capture the main mechanisms of l-serine transport across a range of experimental conditions, using only a minimum number of parameters. Significantly, the model accounted for the different transport mechanisms by varying the mobility of an unloaded transporter, and its predicted effect on tracer accumulation in response to differing concentrations of amino acids at both sides of the plasma membrane. To facilitate biologic interpretation and the estimation of a unique set of model parameters, the transport properties were assumed to be symmetric across the plasma membrane. However, this is not an intrinsic limitation of the transport model, and the introduction of additional parameters may improve representation (19).

In keeping with our initial modeling paradigms (19), l-serine was selected as the experimental amino acid substrate to test the carrier model. l-serine is transported primarily with relatively high affinity by the system L transporter LAT2 and not by LAT1 (10, 11, 15, 16, 18). We therefore propose that our model describes the transport mechanism of the LAT2 transporter. Although substrate inhibition studies have evidenced LAT2 transport activity in human placental MVM, we demonstrate for the first time the expression of the LAT2 protein in this membrane, supporting LAT2 mediated l-serine transport (16, 26, 35). This is a novel finding because LAT1 was thought to be polarized to the MVM (36), whereas LAT2 was suggested to be the dominant system L isoform on the opposing BM of SCT (24). The colocalization of both LAT1 and LAT2 to both SCT plasma membranes therefore suggests that the distribution of these proteins may not be as highly polarized as in other epithelia (6, 9, 37) and that they may work in concert to mediate transplacental amino acid transport.

The agreement of the experimental data to the mixed transport model (in comparison to the obligate exchange model) provides confidence that this is a reliable mechanistic framework to describe LAT2-mediated transport across MVM. This is an important finding as there is reported heterogeneity in terms of its obligatory nature. Although Sewaga *et al.* were unable to *trans*-stimulate l-leucine efflux from preinjected *Xenopus* oocytes transfected with LAT2 inferring facilitated transport (18), others demonstrate *trans*-stimulation of amino acid uptake into preinjected *Xenopus* oocytes transfected with LAT2, interpreted to represent obligate exchange (9, 15, 18). Consistent with the latter, we show that preloading vesicles with l-serine leads to higher intravesicular concentrations of [^14^C]l-serine above nonloaded controls, demonstrating that transport can be *trans*-stimulated. Although this has typically been interpreted as evidence for exchange, it must be borne in mind that a facilitated transporter can also display *trans*-stimulation (*i.e.,* faster return of the carrier because of the mediated efflux of intravesicular substrate, leading to accelerated uptake by speeding up the transport cycle; Fig. 1*B*).

The key factor in determining the contribution of a facilitated transport component was the computational simulation for experiment 4b. The mixed transport model predicted that a high outwardly directed l-serine concentration gradient would elicit overshoot curve; this was not predicted to occur if exchange was fully obligatory. An overshoot curve was subsequently observed experimentally and was similar to the simulated substrate curve with respect to peak height. Overshoot phenomena are reported in other transport systems (38); however, little is known regarding the relationship between the shape of the curve and the characteristics of the underlying transport mechanisms. Computational simulation has therefore driven us to explore these phenomena further, and we interpret the overshoot curve to represent initial uptake by a rapid exchange mechanism (overshoot peak) followed by a decline in uptake (or alternatively increased tracer efflux) mediated by a (slower) facilitated transport component (overshoot decay). This is because in the absence of a facilitated transport route, l-serine would continue to accumulate until reaching a constant concentration determined by the original intracellular amino acid concentration, which was not observed. However, it is important to note that, although facilitated transport may play a role driven by imposed substrate gradients, the dominant mechanism relating to system L-mediated transport is exchange. What mechanism dominates *in vivo* will depend on the transmembrane gradients present, *e.g.,* as long as counter gradients are maintained, then exchange will result in sustained faster uptake.

The overshoot phenomenon was further confirmed by a variety of internalized system L substrates (l-alanine, l-methionine, glycine, d-leucine, l-leucine, and l-phenylalanine). The varying magnitude of the overshoot peaks suggests that the identity of the intracellular amino acid modulates the capacity for l-serine *trans*-stimulation. This supports previous studies illustrating selectivity of exchange by internalized amino acids. For example, l-phenylalanine is an effective substrate for l-tryptophan influx (21), whereas we show that l-phenylalanine is a relatively poor substrate for l-serine influx. This suggests asymmetry for exchange and agrees with previous studies showing that influx and efflux substrate selectivity may not be matched. However, of particular interest was the observation that different internalized system L substrates led to different internal concentrations of l-serine at equilibrium. The reasons for this variability cannot be fully explained at present. However, it does suggest the presence of an unknown parameter mediating this selectivity and may provide one explanation for the underestimation of the experimental serine equilibrium concentrations by the mixed transport model. Interestingly, certain internalized amino acids behaved rather unexpectedly. Both l-proline and MeAIB, classic substrates of the Na^+^-dependent system A transporters but not system L (18, 21, 39, 40), significantly stimulated [^14^C]l-serine influx above nonloaded controls (yet lacked overshoot seen with classic LAT2 substrates). The mechanisms underlying this is unclear; however, a contribution by other transporters cannot be excluded. Furthermore, internal BCH, an amino acid analog typically used to study system L-mediated transport (extracellular BCH inhibits uptake of system L substrates) (16, 26, 41) also stimulated [^14^C]l-serine influx. This indicates that BCH is not only an inhibitor but that it is also an effective substrate for system L transporters, as previously demonstrated (21, 42).

The ability of a range of amino acids to elicit overshoot suggests a common mechanism mediating the transport of l-serine across the MVM. We believe this to be a mechanism that shows characteristics of LAT2-mediated transport, and as indicated by the carrier model fittings, one that displays characteristics of both antiport and uniport mechanisms. If the apparent exchange associated with LAT2 becomes less tightly coupled, then exodus of intravesicular amino acid can occur without concomitant uptake of another amino acid. Such slippage (uncoupled translocator) has been reported for other antiport systems that also allow low levels of uniport function (43, 44). It is possible that slippage represents a function of carrier protein biochemistry that may constitute a physiologic mechanism (44–46), although it is not immediately clear at present what the physiologic relevance of this might be. On the other hand, we cannot fully exclude the existence of an additional (kinetically similar) facilitated l-serine transporter in parallel, although there are no known reports of these transporters on the MVM of human placenta at present. This emphasizes the need to investigate the functional role of these transporters as part of a wider physiologically integrated transport system.

Consistent with previous observations (16), l-serine was transported into MVM vesicles in the absence of *trans*-amino acid, raising the question of how we observe system L activity in the absence of added intravesicular amino acid. We show that zero-*trans* uptake may, in part, be explained by the presence of endogenous amino acids found within MVM vesicle isolates that, provided they are freely exchangeable, may be sufficient to initiate exchange. However, our model shows that the experimental data cannot be explained by perfectly obligatory exchange driven by endogenous substrate, whether their concentrations were low or high (Fig. 3*D*). Furthermore, their collective concentrations (in particular for the system L substrates) were much lower than those determined to drive maximal transporter activation (*e.g.,* overshoot), as set by our experimental conditions. The origin of the observed endogenous amino acids is not clear; however, their relative concentrations follow a similar ranking to previous estimates of free intracellular amino acid concentrations in human placental tissue (34), suggesting some degree of procedural carryover. Moreover, whether the measured concentrations of endogenous amino acids *in vitro* are a reliable representation of concentrations at the fluid tissue interface in vivo is uncertain. Unstirred layers at the membrane surface of the MVM may lead to concentration gradients within the glycocalyx such that concentrations of solutes are different to that in the bulk solution. However, although potentially affecting kinetic parameters, it does not alter our findings in relation to the transport mechanism.

In summary, our findings suggest that exchange and facilitated transporters are 2 ends of a spectrum and that where they are placed on this spectrum is determined by the speed of return of the unloaded carrier. The integration of computational modeling with amino acid transport studies has therefore generated new mechanistic insights into the different modes of amino acid transport. Modeling has allowed us to predict how transporters should act based on our understanding, to design experiments that will specifically test our understanding, and to interpret the results of these experiments. An iterative cycle of model predications, experimental validation, and model refinement will therefore lead to an improved quantitative understanding of the interactive mechanisms underlying amino acid transfer as a biologic system in both health and disease.

## Abbreviations

BCH: 2-aminobicyclo[2.2.1]heptane-2-carboxylic acid
BM: basal plasma membrane
EX: amino acid exchanger
FT: facilitated transporter
IVB: intravesicular buffer
LAT: L-type amino acid transporter
MeAIB: *α*-methylaminoisobutyric acid
MVM: microvillous plasma membrane
SCT: syncytiotrophoblast
